# Lessons from the model gut Bacteroidales *Bacteroides fragilis* and *Bacteroides thetaiotaomicron* and future opportunities

**DOI:** 10.1128/jb.00346-25

**Published:** 2025-10-16

**Authors:** Laurie E. Comstock

**Affiliations:** 1 Duchossois Family Institute University of Chicago 2462, Chicago, Illinois, USA; 2 Department of Microbiology, University of Chicago 2462, Chicago, Illinois, USA; Geisel School of Medicine at Dartmouth, Hanover, New Hampshire, USA

**Keywords:** mcirobiota, microbiome, Bacteroidales, Bacteroides

## Abstract

Bacteroidales is an order of bacteria that includes members that colonize the human gut, oral cavity, cow rumen, and other host-associated environments. Most humans become colonized with gut Bacteroidales species relatively soon after birth and later become colonized at high density with numerous diverse species. Bacteroidales strains often persist in the human gut for decades where they extensively evolve, acquiring point mutations, prophage, mobile plasmids, and integrative conjugal elements, making each person’s gut Bacteroidales strains highly personalized. Much of what we have learned about basic biological properties of gut Bacteroidales comes from analyses of two species, *Bacteroides fragilis* and *Bacteroides thetaiotaomicron*, which were studied for different reasons. Three decades ago, there was only one human gut Bacteroidales genus recognized, the *Bacteroides*, into which all human gut Bacteroidales species were classified. Today, the human gut Bacteroidales number over 50 species with more than 14 genera and at least seven families. Studies of *B. fragilis* and *B. thetaiotaomicron* have provided a wealth of information of basic processes of these gut symbionts, many of which are generally applicable to other species of gut Bacteroidales. In this review, I provide a historical perspective as to why these two species have served as models, as well as some of the biological processes learned from studies of these two species. Finally, I discuss why present and future analyses of the gut Bacteroidales have expanded beyond these two model organisms.

## INTRODUCTION

In the 30 years since I began studying the gut Bacteroidales, there has been a huge transformation in this field due to increased awareness of the importance of the gut microbiota to human health and disease. The microbes of the human gut microbiota are studied not only by microbiologists but also computational biologists, ecologists, evolutionary biologists, immunologists, developmental biologists, and in the fields of metabolomics, medicine, and other disciplines, making these once largely ignored organisms now the subject of intensive investigation.

Studies of *Bacteroides fragilis* and *Bacteroides thetaiotaomicron* have provided much of what we know of the biology of gut Bacteroidales. In 1990, only a handful of genes of any gut Bacteroidales had been sequenced; most of those involved in antibiotic resistance (1–8). Most investigators in this field in the mid-1990s were those, like me, who were studying *B. fragilis* as an opportunistic pathogen, which occurs if they gain access to extraintestinal sites. Although other Bacteroidales species are isolated from extraintestinal infections, *B. fragilis* is one of the most frequently isolated anaerobic opportunistic pathogens, contributing to the formation of intra-abdominal abscesses, brain and lung abscesses, bone infection, diabetic foot infections, and other clinical manifestations (9). Therefore, studies of *B. fragilis* provided much of the fundamental knowledge about the gut Bacteroidales prior to 2000. The bulk of basic studies of this species were performed using three strains, NCTC 9343, 638R (10, 11), and YCH46 (12).

## TAXONOMIC CHANGES THROUGH THE YEARS

**Fig 1:**
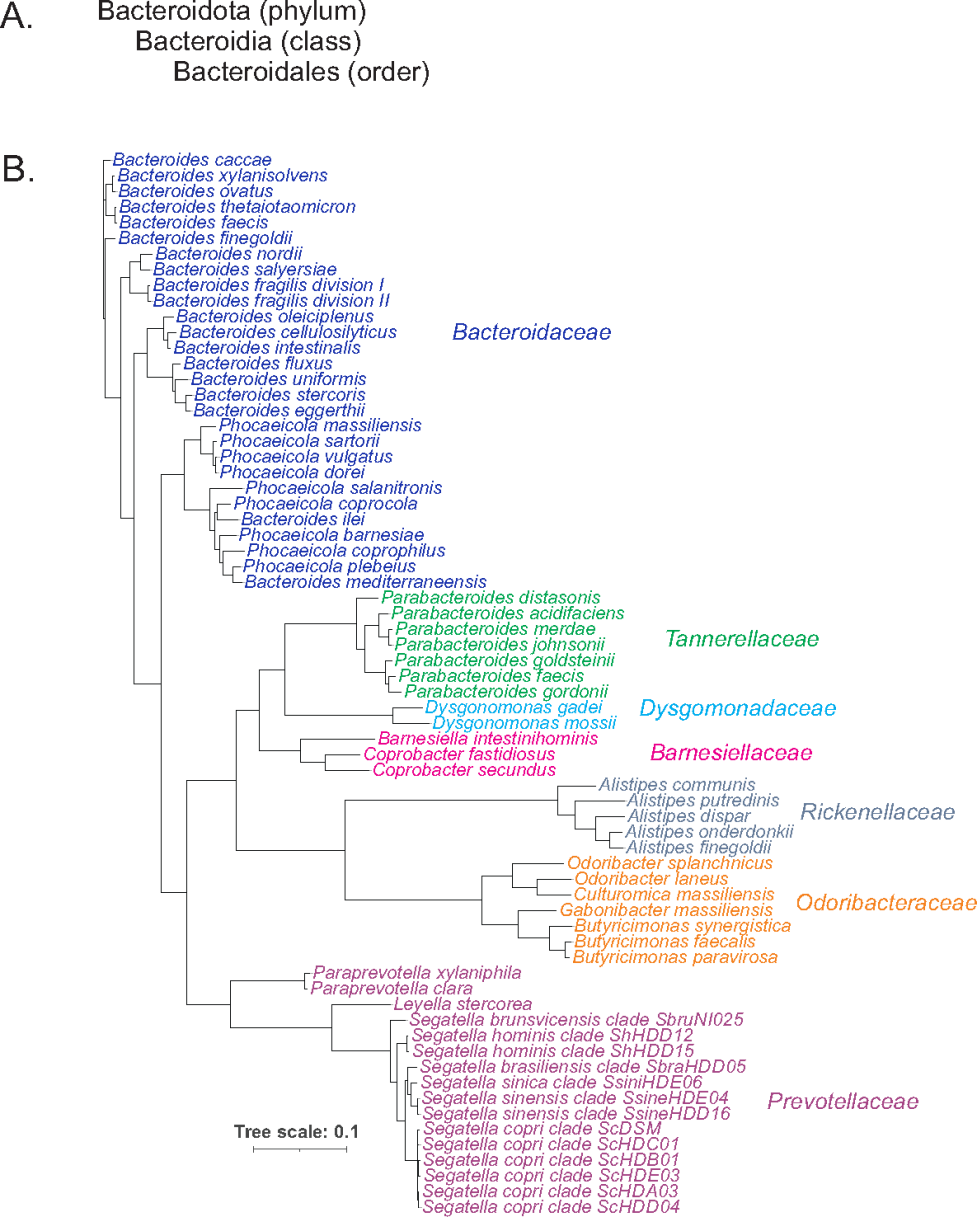
(**A**) The phylum, class, and order into which gut Bacteroidales are contained. (**B**) Phylogenetic tree showing many human gut Bacteroidales species. The tree is based on analyses of 25 core genes. The colors show eight distinct families.

The *B. fragilis*-centric view in the early decades is exemplified by the taxonomy at the time, where most gut Bacteroidales, and even some oral and ruminal Bacteroidales, were considered subspecies of *B. fragilis* or clinically clustered together as the *B. fragilis* group. For example, *B. fragilis* subspecies *distasonis* was the nomenclature for what was designated in 1976 as *Bacteroides distasonis* (13). Then in 2006, it was reclassified as a separate genus designated “*Parabacteroides*” (14) with at least four other species in this genus and belonging to a distinct family, *Tannerellaceae*. Taxonomic redesignation combined with the discovery of new species continues, with a significant reclassification occurring in 2021 that placed *Bacteroides vulgatus* and *Bacteroides dorei* into the genus *Phocaeicola* along with several other gut species previously designated as *Bacteroides* (15). Similarly, the large *Prevotella* genus that included various gut species as well as those of the oral cavity, vagina, cow rumen, and other sites was reclassified into seven distinct genera (16) including *Segatella* (17), which contains the abundant gut Bacteroidales species prevalent in non-industrialized human populations, *Segatella copri*. Recently, *B. fragilis*, which has been known for 25 years to form two phylogenetic clades (18–23), was proposed to be reassigned as two species (24, 25). Therefore, with the advent of genomics and refined phylogenetic tools, these bacteria have now been properly classified and include previously unrecognized genera such as *Alistipes* (26), *Barnesiella* (27), *Odoribacter* (28), and *Butyricimonas* (29), among others (Fig. 1).

## THE RISE OF *B. THETAIOTAOMICRON* VPI-5482 AS A MODEL ORGANISM

Unlike *B. fragilis*, where three distinct strains have been heavily studied, a single *B. thetaiotaomicron* strain, VPI-5482, has served as a model. Although *B. fragilis* was studied for its pathogenic potential, *B. thetaiotaomicron* has been studied largely as a gut symbiont. Prior to 1990, only a few investigators such as Abigail Salyers, Jeffrey Gardner, Tracey Wilkins, and a few others were studying biological processes of *Bacteroides* to understand their capacity to colonize the human gut, that is, as gut symbionts. This was a time when the terms microbiota and microbiome were not used to describe human gut microbial communities, in favor of the term “microflora.” I will highlight many of Salyers’ and colleagues’ seminal discoveries below, but here I recount what I witnessed that led to the use of *B. thetaiotaomicron* VPI-5482 as a model organism.

*B. thetaiotaomicron* is not the most abundant *Bacteroides* species in humans, and neither its prevalence nor abundance accounts for why it is so well-studied. In the mid-1970s, Salyers was studying various gut Bacteroidales including *B. thetaiotaomicron* VPI-5482, isolated at the anaerobe lab at Virginia Polytechnic Institute where she was a post-doctoral fellow with Tracy Wilkins. Salyers, Wilkens and colleagues (30) showed that this strain was able to grow on numerous host and dietary polysaccharides (PS), one of the first analyses showing that these bacteria are dietary PS-degraders (glycophiles). Although Salyers’ subsequent studies also analyzed glycan and carbohydrate utilization by other *Bacteroides* species, she selected *B. thetaiotaomicron* VPI-5482 as her model strain for molecular analyses. An important paper published in 1989 described the screening of a transposon bank of *B. thetaiotaomicron* VPI-5482 for mutants unable to grow on various monosaccharide and PS sources (31). One of the characterized mutants was unable to utilize fucose as a carbon source. One of the first manuscripts analyzing gut symbionts from Jeffrey Gordon’s lab showed that fucosylation of proteins on host epithelial cells requires the gut microbiota (32). In this study, the authors showed that *B. thetaiotaomicron* was able to restore surface fucosylation to previously germ-free mice, but the *B. thetaiotaomicron* fucose utilization deficient mutant that his lab acquired from Salyers did not induce fucosylation of epithelial cells. Due to the genetic tools created in *B. thetaiotaomicron* VPI-5482 and the accumulated knowledge of this strain by Salyers and colleagues, the Gordon Lab continued to use *B. thetaiotaomicron* VPI-5482 as their model *Bacteroides*. It was the wealth of studies from the Gordon Lab using *B. thetaiotaomicron* VPI-5482, including the first published genome sequence of a gut Bacteroidales (33), the creation of a mutant strain for counterselection of double crossover recombination for DNA deletions and insertions (34), and the creation of the first INSeq bank (35) that led to the continued use of this strain as the model gut *Bacteroides*. In addition, the Gordon Lab produced many prolific *Bacteroides* investigators, many of whom continued to use *B. thetaiotaomicron* VPI-5482 as their model strain. Therefore, it was the initial seminal discoveries from Salyers combined with the high-profile studies from the Gordon Lab and the resources they created that largely propelled *B. thetaioaomicron* VPI-5482 as the model gut Bacteroidales strain. In addition, studies from the Gordon Lab from the early 2000s analyzing the effects of the microbiota on host processes (36) and that linked the microbiota with obesity (37–40) greatly contributed to the rapid increase in interest in the gut microbiota among scientists of diverse disciplines.

## FIRST GENOME SEQUENCES

Several *B. fragilis* investigators, including myself, met at the 1999 American Society for Microbiology General Meeting in Chicago to strategize how to encourage the National Institutes of Health (NIH) to support funding a *B. fragilis* whole genome sequence project. However, as the genomes of many pathogenic bacteria had not yet been sequenced and the importance of gut symbionts to human health was not yet fully appreciated, *B. fragilis* was not prioritized. The next 8 years brought a tremendous transformation in the characterization of host-associated microbial communities (41–43) and demonstrations of their importance to human health, so that not only were these bacteria prioritized, but the NIH Human Microbiome Project (HMP) was launched in 2007 (https://commonfund.nih.gov/hmp), with a similar large program, Metagenomics of the Human Intestinal Tract (MetaHIT), funded by the European Commission (https://www.gutmicrobiotaforhealth.com/metahit). Fortunately, the sequences of a few gut *Bacteroides* genomes were published in those intervening years, the first being *B. thetaiotaomicron* VPI-5482 published in 2003 by the Gordon Lab (33) followed in 2004 by *B. fragilis* YCH46 completed by a Japanese consortium (44) and then in 2005 the genome sequence of *B. fragilis* NCTC 9343 by the Sanger Institute, Wellcome Trust, and UK investigators (45), although sequences of this genome were made publicly available as they were generated beginning in 2001. In 2007, the Gordon Lab published genome sequences of two other gut Bacteroidales species, now known as *Phocaeicola vulgatus* ATCC 8482 and *Parabacteroides distasonis* ATCC 8503 (46). Around 2012, the genome sequences of several more Bacteroidales strains were released as part of the HMP, including 26 Bacteroidales strains isolated from my lab and sequenced by the Broad Institute (CL strains). The availability of the genome sequences of diverse Bacteroidales species allowed for an acceleration in scientific discovery of species beyond *B. fragilis* and *B. thetaiotaomicron*. Below, I highlight some of the discoveries made studying *B. fragilis* and *B. thetaiotaomicron*, including those from the first genome sequences of these species. I highlight basic properties of these bacteria but do not discuss studies showing how these bacteria affect the host, which is itself a very large field of investigation. Although I cannot include all the interesting discoveries through the years, I will focus on a few well-studied functions/processes, especially those that differ from those of well-studied *Pseudomonadota* species.

## ANTIBIOTIC RESISTANCE

As *B. fragilis* is an important opportunistic pathogen, its resistance to antibiotics has been a topic of study since the 1950s (47–49), and resistance profiling studies dominated the *Bacteroides* literature through the 1970s. Antibiotic resistance monitoring of species isolated from extraintestinal sites has continued through the decades (50–52). *B. fragilis* and closely related species are largely resistant to aminoglycosides and non-carbapenem β-lactams. In the 1950s, prior to the clinical use of tetracycline, most strains were sensitive, but studies from the 1970s and 1980s were revealing that resistance to tetracycline and other antibiotics was increasing and was transferable to other strains in the laboratory (11, 53–59). Today, *Bacteroides* strains remain largely sensitive to metronidazole and carbapenems, which are used therapeutically along with piperacillin/tazobactam. As carbapenems and metronidazole are some of the last line antibiotics for *Bacteroides*, their resistance genes have not been used by researchers as selectable markers for basic science studies. Although a high percentage of strains are resistant to erythromycin, tetracycline, and cefoxitin, few strains are resistant to all three, and these antibiotic resistance genes, as well as a non-Bacteroidales chloramphenicol resistance gene (*cat*) (60), are commonly used for genetic manipulation of Bacteroidales species (discussed later in this review).

## INTEGRATIVE MOBILE GENETIC ELEMENTS

**Fig 2:**
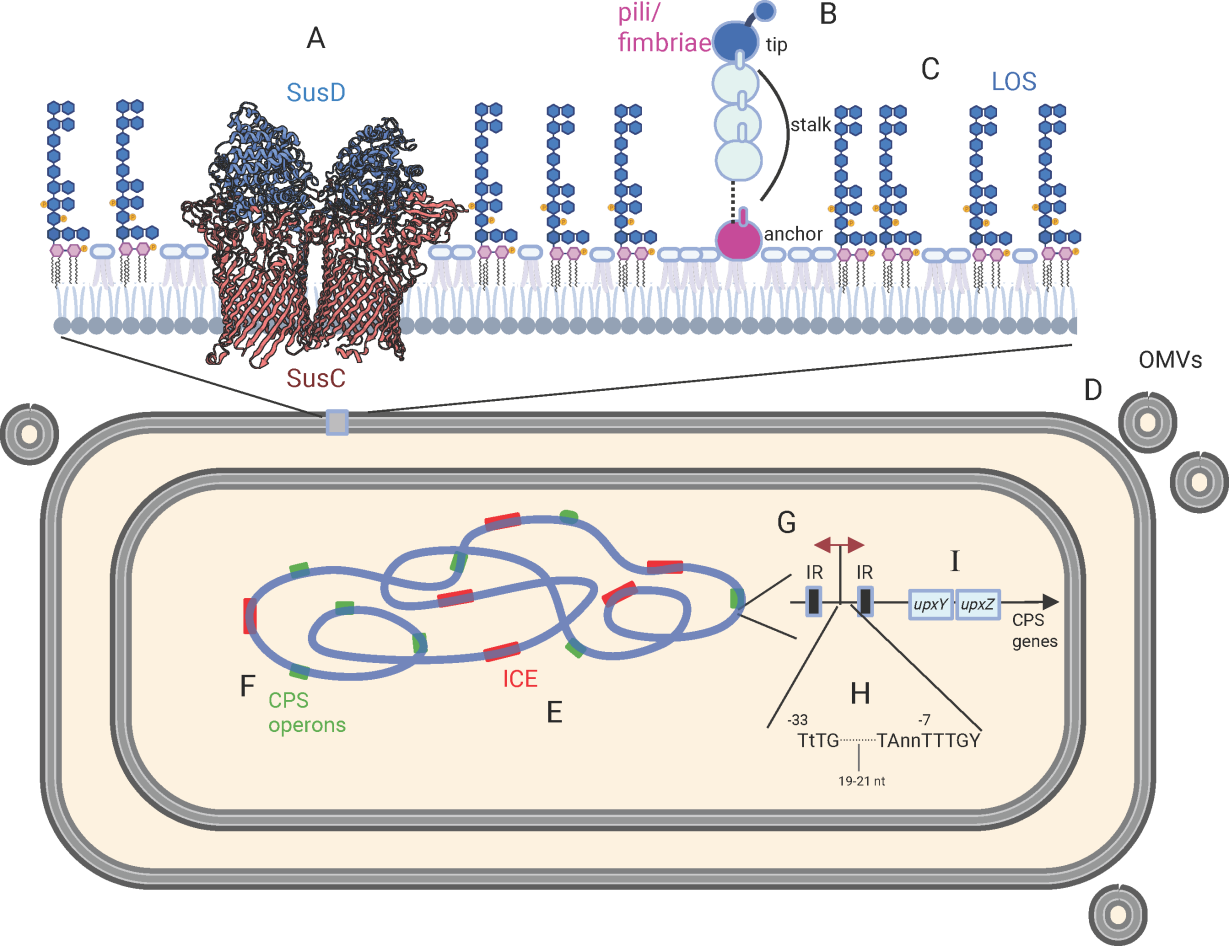
Depiction of some of the cellular structures and processes discussed. (**A**) Two SusCD family complexes (fructo-oligosaccharide transporter BT 1762-63) from 6Z8I | pdb_00006z8i. The external SusD ortholog is colored blue, and the SusC ortholog spanning the outer membrane is colored maroon. (**B**) The type V pili/fimbriae of the Bacteroidota showing the three distinct pilin subunits. The anchor pilin retains the N-terminal lipidation that anchors into the outer membrane. The tip pilin often has an extra C-terminal ligand binding domain. (**C**) A schematic representation of the LOS of *B. thetaiotaomicron* VPI-5482. Blue hexagons represent monosaccharides, and yellow shapes represent phosphate groups. The lipid A is shown with five acyl chains extending from the glucosamine residues (pink). (**D**) Outer membrane vesicles released from the bacterial cell. (**E**) Red rectangles represent ICE that are scattered throughout gut Bacteroidales genomes. (**F**) Green rectangles represent the numerous CPS biosynthesis loci of gut Bacteroidaceae species. (**G through I**) Schematic of the region upstream of the CPS loci showing (**G**)inverted repeat regions (IR) flanking the promoter region that undergoes inversion. (**H**) The consensus σ^70^ promoter region. (**I**) Two regulatory gene families (*upxY* and *upxZ*) that are often the first two genes of CPS biosynthesis loci and regulate transcriptional antitermination (UpxY) and cross-locus transcriptional inhibition (UpxZ). Illustration made with Biorender.

The early 1980s was a time when genetic elements now known as integrative and conjugative elements (ICEs) were being discovered in diverse bacteria including *Bacteroides* (61, 62). These elements were first designated as conjugative transposons (63), but as similar elements were discovered in other bacteria, several designations were used for the same type of conjugative element (64, 65). In 2002, the designation of integrative and conjugative element was proposed as a unifying term for all these similar elements (66). Nearly all of the early studies from the 1980s and 1990s of conjugative transposons/ICE of the gut Bacteroidales came from Salyers’ lab. One of the model ICE studied in her lab was CtnDOT, a 65 kbp element containing genes encoding both tetracycline and erythromycin resistance (67). Studies showed that ICE contain all the genes for their conjugal transfer, including integrases, excisionases, proteins involved in synthesis of the conjugative apparatus (Tra proteins), and regulatory proteins (68–70). With the advent of genomics and metagenomics, the vast extent of ICE in the gut Bacteroidales is now appreciated (Fig. 2E), as is the rapid dissemination of these elements to other Bacteroidales species in the human gut and across human populations (71–74). These elements have been shown to harbor cargo genes that are involved in bacterial antagonism (75, 76), that protect bacteria from antagonistic attacks (77), that enable the utilization of additional dietary PSs (78), and that allow the acquisition of critical vitamins (79), among other functions. The complex regulatory network governing the transfer of CtnDOT following exposure to low levels of tetracycline has been elucidated (reviewed [80]). However, numerous Bacteroidales ICE lack similar regulatory genes, and their transfer likely involves distinct regulatory systems, a topic ripe for future investigation.

## PS UTILIZATION LOCI (PUL)

The ability of the gut Bacteroides to utilize dietary plant PS and host glycosylated molecules as carbon sources was first demonstrated in the 1970s (30, 81–83). In a series of studies from Salyers and colleagues from the late 1980s to early 2000s revealed the proteins involved in the utilization of starch in *B. thetaiotaomicron* VPI-5482 (84–91). These proteins were shown to be encoded by adjacent genes that they named Sus for “starch utilization system.” Proteins designated SusC and SusD were shown to interact with each other and function with other *sus*-encoded outer surface starch binding proteins and surface glycoside hydrolases (GHs) for starch and maltooligosaccharides utilization. Additionally, they showed these loci are regulated by SusR and MalR, which mediate maltose-dependent regulation of *sus* genes (92, 93).

It was not until the *B. thetaiotaomicron* VPI-5482 genome was sequenced that the large number of loci similar to *sus* was revealed. The genome sequence revealed an extensive number of adjacent *susC* and *susD* orthologs, often surrounded by genes encoding glycosyl hydrolases, PS lyases, and regulatory proteins (33). The loci were termed PUL for PS utilization loci (94) and numbered 88 in the *B. thetaiotaomicron* VPI-5482 genome with 246 glycolytic enzymes (33). This began a new era of studies directed at identifying which PUL(s) are involved in the utilization of which dietary PS or host glycan, and how bacteria sense these nutrients to induce expression (95–98). In addition, studies in *B. thetaiotaomicron* VPI-5482 yielded a more detailed understanding of the SusCD complex (99, 100) (Fig. 2A) as well as functional and structural analyses of many GHs that are too numerous to cite here. Similar studies in other Bacteroidales species revealed differences in the ability to utilize various PS, with some species preferentially using dietary PS over mucin O-glycans (101) and others incapable of utilizing mucin O-glycans (97, 102, 103). This remains one of the most active areas of Bacteroidales research, and knowledge from these studies is being harnessed for assembling communities for gut microbiome restoration.

## CPS AND GLYCOPROTEINS

Capsular PS (CPS) were originally studied in *B. fragilis* due to their involvement in the formation of intra-abdominal abscesses (104). When I started working with *B. fragilis* in 1995, it was thought that strain NCTC 9343 produced two distinct CPS (PSA and PSB) (105), the structures of which were both elucidated (106). My initial goal was to identify, clone, sequence, and delete the PSA and PSB loci of *B. fragilis* so that their involvement in abscess formation could be demonstrated using isogenic mutants. In the pre-genomics era, the sequencing of these large biosynthesis loci was a time-consuming endeavor. In one of our first papers describing one of these PS biosynthesis loci, a reviewer questioned why we were studying the CPS loci of *B. fragilis*, stating that everything we needed to know about CPS biosynthesis could be learned from *Escherichia coli*. Over the first 6 years of study, we showed that strains of *B. fragilis* produce not two, but eight, distinct CPS (Fig. 2F), designated PSA-PSH (107). We showed that the promoter regions of seven of these eight loci undergo DNA inversion leading to phase variation (107) (Fig. 2G), that these DNA inversions were mediated by a single global DNA invertase (108), and that there was a second layer of regulation mediated by unique UpxY/UpxZ proteins (Fig. 2I) that mediate transcriptional antitermination and cross-locus repression of transcriptional antitermination (109–111), properties not present in *E. coli*. The subsequent genome sequence of *B. thetaiotaomicron* VPI-5482 revealed that it also has eight CPS loci (CPS1-8), many of which have invertible promoters. The synthesis of multiple phase variable CPS is a common feature of gut Bacteroidales, a property not shared with oral Bacteroidales species (112). Elucidation of the functional importance of these CPS continues to be a heavily investigated area of research.

It was through studies of the CPS of *B. fragilis* that a hybrid mammalian-like protein was discovered that mediates direct conversion of salvaged fucose into guanosine diphosphate (GDP)-fucose for incorporation into bacterial glycans (113). Studies of this enzyme led to the discovery that these species, and species throughout the Bacteroidota phylum, have a general protein glycosylation system that is essential for viability of these bacteria (114, 115). Protein glycosylation remains an understudied area of investigation with questions regarding both their biosynthesis and function remaining to be addressed.

## DNA INVERSIONS

It was through studies of the CPS biosynthesis loci of *B. fragilis* that DNA inversions were first identified in the gut Bacteroidales (107). Genome sequences of *B. thetaiotaomicron* VPI-5482 (33), *B. fragilis* YCH46 (44), and *B. fragilis* 9343 (45) revealed the extreme number of regions in these genomes that undergo inversion and that affect other molecules including surface proteins (116–118), EPS loci (119) restriction modification systems (45, 120, 121), and SusCD orthologs (122), among others. These invertible regions include not only promoter regions but larger DNA segments that move genes into expression loci and also include within-gene inversions (123). Current studies are addressing how the ability of these bacteria to create distinct phenotypic populations allows them to better survive and compete in the gut.

## LPS AND LOS

Studies from the 1990s showed that most *Bacteroides* species synthesize a lipooligosaccharide (LOS), with the exception being *P. vulgatus*, then designated as a *Bacteroides*, which was shown to synthesize an O-antigen (124). It was only within the last 3 years that the full structures of the lipopolysaccharide (LPS) of *P. vulgatus* strain mpk (125) the LOS of *B. thetaiotaomicron* VPI-5482 (126) and the LOS of *B. eggerthii* 1_2_48FAA (127) have been elucidated. The LOS of *B. thetaiotaomicron* is a highly charged and branched structure comprised of 15 sugars (Fig. 2C). The LPS of *P. vulgatus* mpk comprises a seven-sugar core with a laddering O-antigen comprised only of rhamnose and mannose. Despite the advances in structural analyses, there is still a paucity of analyses of the biosynthesis loci and genes involved in the production of the LOS/LPS glycans (128–132). The lipid A component was shown decades ago to have weak endotoxin activity (133, 134) as would be expected of gram-negative bacteria that reach extremely high densities in the mammalian gut. Early structural studies (135–137) supported later by full structural studies (125–127) showed that the lipid A has only four or five acyl chains extending from the glucosamine residues compared to the six acyl chains typical of *E. coli* lipid A. In addition, only one of the glucosamine residues is phosphorylated, another feature that reduces its endotoxin activity. The single phosphate on the lipid A was shown to result from a dephosphorylation reaction and protects *Bacteroides* species from host antimicrobial peptides during inflammation (138).

## SURFACE LIPOPROTEINS, LES, CLOSTRIPAINS, AND OMVS

As opposed to *E. coli* where lipoproteins are predominantly anchored to the inner leaflet of the outer membrane or on the outer leaflet of the inner membrane, most Bacteroidales lipoproteins are flipped to the surface of the bacterium. The signal peptidase II (SpII) signal sequence is an easily identifiable N-terminal sequence that dictates cleavage and lipidation of the cysteine residue at the cleavage site (139). A lipoprotein export signal (LES) was identified and found to be comprised of several D/E residues following the lipidated cysteine and reported as K-(D/E)_2_ or Q-A-(D/E)_2_ (140). Lipoproteins with the LES are exported to the outer surface by an as-yet undescribed protein(s). A study in *B. fragilis* showed that a surface clostripain-like protease termed fragipain (Fpn) cleaves and activates the *B. fragilis* enterotoxin BFT (141). This protease was also shown to cleave and release numerous surface lipoproteins (142, 143) including a secreted antibacterial protein (144). Orthologs of Fpn are conserved in species of the Bactoroidota phylum (143), and analyses in *Phocaeicola dorei* show that its surface clostripain, termed doripain, cleaves, and activates two distinct secreted antibacterial toxins (145, 146)

Surface lipoproteins can also be released from *Bacteroides* in outer membrane vesicles (OMVs) where they mediate processes that affect the host (147–154) and other bacteria (155–159), influencing both cooperative and competitive interactions. Studies in both *B. fragilis* and *B. thetaiotaomicron* have shown that *Bacteroides* produce copious amounts of OMVs (Fig. 2D) and that surface proteins with the LES domain can also be packaged into OMVs (160).

## TYPE V PILI

It was many years after publication of the first genome sequences of *B. thetaiotaomicron* and *B. fragilis* that the fimbriae/pili of these bacteria were identified. This lag in their identification is due to a lack of sequence similarity to characterized fimbriae proteins of other bacteria. An early study reported the N-terminal sequence of a fimbrial protein (161), and later our lab characterized a phase-variable locus associated with an adherent/aggregative phenotype encoding an ORF matching the N-terminally sequenced protein (116). However, as we were not able to show any typical fimbriae surface appendages using antisera to several of the subunits, we could not conclude that these outer surface proteins were *bona fide* fimbriae. Studies in *Porphyromonas gingivalis*, a related oral pathogenic Bacteroidales species, led investigations of the fimbriae of Bacteroidales (162–164). However, it was not until a study reporting the structures of 20 Bacteroidota pilins that this new group of pili was revealed, which are distinct from all previously reported pili. Type V pili are comprised of three structural pilin proteins: a tip pilin, typically with a C-terminal ligand binding domain, a stalk pilin that is the repeated subunit comprising the bulk of the fimbriae, and an anchor pilin that anchors the pilin to the cell (Fig. 2B). These pilins begin as outer surface lipoproteins that are proteolytically cleaved by the surface clostripain at their N-terminus during assembly, creating a hydrophobic groove that binds the C-terminus of the incoming pilin. The lipid moiety of the anchor protein tethers the pilin to the cell. A recent study showed that diversity-generating retroelements are prevalent in *Bacteroides* and are leading to increased diversification of the tip pilins (165), likely modifying the binding domain. We still know very little about what these fimbriae bind, although some have lectin domains suggesting they facilitate binding to bacterial, dietary, or host glycans.

## PROMOTER SEQUENCE

The canonical *E. coli* promoter does not support transcription in *Bacteroides* (60). The consensus σ^70^ promoter sequence was identified in *B. fragilis* by comparative analyses of regions upstream of several sequenced genes of *B. fragilis* (166). This promoter sequence is relatively conserved in other members of the Bacteroidota phylum (167–169), and its identification greatly accelerated genetic analyses of these bacteria. Unlike the canonical −10 and −35 promoter sites of *E. coli*, *Bacteroides* have a −33 and −7 site, with the consensus sequence of the −7 site TANNTTTGY (166) (Fig. 2H). Subsequent studies of the σ^70^ protein that recognizes the promoter showed that it lacks the N-terminal 1.1 region (170), an unstructured acidic N-terminal domain present in essential sigma factors of other organisms. *Bacteroides* species also have an extensive number of alternate sigma factors of the extracytoplasmic function (ECF) type (50 in *B. thetaiotaomicron* and 43 in *B. fragilis* 9343) (33, 45). These sigma factors are typically paired with antisigma factors that sequester the factor until a signal is detected, causing the antisigma factors to release the sigma factor to transcribe its regulon. This extensive number of such ECF sigma factors allows these bacteria to respond to numerous nutrients and other factors in their changing environment.

## OXIDATIVE STRESS RESPONSE

One of the properties that makes *Bacteroides* relatively easy to study is the fact that most species are aerotolerant, withstanding extended periods of exposure to air. The bulk of analyses of the oxidative stress response was performed in the Smith lab using *B. fragilis* 638R (171–175). These studies showed that *Bacteroides* have a global regulator, OxyR, that activates its regulon almost immediately after exposure to oxygen or hydrogen peroxide. The oxidative stress response includes catalase and six peroxidases. Another transcriptional regulator named BmoR was shown to help regulate the cellular balance of thiol/disulfide, confirming its role in the oxidative stress response (176). In addition to the ability of *Bacteroides* species to mount a robust oxidative stress response, some Bacteroidales species can grow in nanaerobic concentrations of oxygen (177–180). Oxygen diffusing from the intestinal tissue creates an oxygen gradient that bacteria at the interface with the host are exposed to and can use as a terminal electron acceptor (180, 181). *B. fragilis* has constitutively expressed genes encoding the cytochrome *bd* oxidase complex, which can receive electrons from the menaquinone pool during nanaerobic respiration (179). Although several Bacteroidales species and most of the Bacteroidaceae family are aerotolerant, other gut Bacteroidales are less so. Although there are few studies in other Bacteroidales families, the viability of *S. copri* is quickly reduced in the presence of atmospheric concentrations of air (182). Similar studies of other gut Bacteroidales species are certainly warranted as research is expanding to include these diverse species.

## GENETIC TOOLS AND THEIR PROGRESSION

Genetic tools to manipulate *Bacteroides* have existed for decades based largely on plasmids and elements from *B. fragilis* and *B. thetaiotaomicron* including vectors to make transposon banks (183), multicopy plasmids for recombinant gene expression (60, 184), and for construction of insertion and deletion mutants (185, 186). These tools have been extensively refined through the years with the construction of a *tdk* mutant of *B. thetaiotaomicron* VPI-5482 in 2008 that allowed for genetic manipulation of this strain with counterselection (34), with improved transposon vectors (187), TnSeq (InSeq) vectors (35, 188), barcoded transposon sequencing vectors (189), constructs with inducible promoters (190, 191), and in 2019 with vectors for genetic engineering in Bacteroidaceae species with counterselection not requiring a mutant background strain (192, 193). These tools have made genetic-based analyses of *Bacteroides*, *Phocaeicola*, and some *Parabacteroides* strains relatively straightforward. In addition, CRISPR-based gene editing tools have been created for genetic manipulation of gut Bacteroidaceae (194–200). Although transposon libraries have been created in *B. fragilis* strains, it is much more difficult to make robust banks in this species compared to most *B. thetaiotaomicron* strains and other Bacteroidaceae species and typically requires numerous matings to obtain a substantial number of mutants for screening. In our extensive experience making transposon banks and deletion mutants in various *B. fragilis* strains, we have found that a very low-density *B. fragilis* culture (OD_600_ less than 0.05 after 3 hours growth) typically results in better conjugal transfer. Other modifications and tools to improve genetic work in *B. fragilis* have been recently reported (201).

## STUDIES OF DIVERSE GUT BACTEROIDALES SPECIES AND STRAINS

Although *B. fragilis* continues to be studied as an opportunistic pathogen and *B. thetaiotaomicron* VPI-5482 will continue to serve as a model gut symbiont, studies have expanded well beyond these two species. The reason for this shift is due to many factors. First, each person has a distinct microbiota, and the species that are present are often different from person to person. In industrialized populations, *P. vulgatus* and *Bacteroides uniformis* are some of the most abundant and prevalent Bacteroidales species, yet they are not nearly as well-studied as *B. thetaiotaomicron*. Second, the creation of vectors allowing relatively easy mutant construction in diverse Bacteroidaceae and some *Parabacteroides* strains eliminates the need to use the *B. thetaiotaomicron tdk* mutant strain for genetic manipulations. Also, the accessory genomes of these bacteria are large, with only about 70%–75% of genes part of the core genome of a given species. Therefore, genotypic and phenotypic studies in one Bacteroidales strain are not always representative of that species as a whole. I have spent the bulk of my career studying the 25%–30% of non-core genes which encode surface and secreted molecules that mediate interactions with other bacteria and the host, CPS biosynthesis enzymes, antibacterial toxins, phase-variable fimbriae, restriction modification systems, and other cargo genes carried on mobile genetic elements. The relatively cheap cost of whole genome sequencing has eliminated barriers in analyses of diverse strains and species. Also, the identification of new species of gut Bacteroidales, some of which dominate in non-industrialized human populations and differ in many fundamental properties from the Bacteroidaceae, is increasing studies in diverse human gut Bacteroidales.

## References

[1] Rasmussen JL, Odelson DA, Macrina FL. 1987. Complete nucleotide sequence of insertion element IS4351 from Bacteroides fragilis. J Bacteriol 169:3573–3580. doi:10.1128/jb.169.8.3573-3580.1987 3038844 PMC212434

[2] Rasmussen JL, Odelson DA, Macrina FL. 1986. Complete nucleotide sequence and transcription of ermF, a macrolide-lincosamide-streptogramin B resistance determinant from Bacteroides fragilis. J Bacteriol 168:523–533. doi:10.1128/jb.168.2.523-533.1986 3023281 PMC213512

[3] Smith CJ. 1987. Nucleotide sequence analysis of Tn4551: use of ermFS operon fusions to detect promoter activity in Bacteroides fragilis. J Bacteriol 169:4589–4596. doi:10.1128/jb.169.10.4589-4596.1987 2820936 PMC213826

[4] Hill RT, Parker JR, Goodman HJ, Jones DT, Woods DR. 1989. Molecular analysis of a novel glutamine synthetase of the anaerobe Bacteroides fragilis. J Gen Microbiol 135:3271–3279. doi:10.1099/00221287-135-12-3271 2576872

[5] Thompson JS, Malamy MH. 1990. Sequencing the gene for an imipenem-cefoxitin-hydrolyzing enzyme (CfiA) from Bacteroides fragilis TAL2480 reveals strong similarity between CfiA and Bacillus cereus beta-lactamase II. J Bacteriol 172:2584–2593. doi:10.1128/jb.172.5.2584-2593.1990 2110145 PMC208901

[6] Rasmussen BA, Gluzman Y, Tally FP. 1990. Cloning and sequencing of the class B beta-lactamase gene (ccrA) from Bacteroides fragilis TAL3636. Antimicrob Agents Chemother 34:1590–1592. doi:10.1128/AAC.34.8.1590 2121094 PMC171878

[7] Goodman HJ, Woods DR. 1990. Molecular analysis of the Bacteroides fragilis recA gene. Gene 94:77–82. doi:10.1016/0378-1119(90)90470-c 2227455

[8] Nakayama K. 1990. The superoxide dismutase-encoding gene of the obligately anaerobic bacterium Bacteroides gingivalis. Gene 96:149–150. doi:10.1016/0378-1119(90)90357-w 2265754

[9] Bubeck Wardenburg J. 2021. Bacteroides and abscesses. In Engleberg N, DiRita V, Imperiale M (ed), Schaechter’s Mechanisms of Microbial Disease. Lippincott Williams & Wilkins.

[10] Stiffler PW, Keller R, Traub N. 1974. Isolation and characterization of several cryptic plasmids from clinical isolates of Bacteroides fragilis. J Infect Dis 130:544–548. doi:10.1093/infdis/130.5.544 4607626

[11] Privitera G, Dublanchet A, Sebald M. 1979. Transfer of multiple antibiotic resistance between subspecies of Bacteroides fragilis. J Infect Dis 139:97–101. doi:10.1093/infdis/139.1.97 108340

[12] Ono T, Akimoto S, Kinouchi T, Kataoka K, Ohnishi Y. 1994. Cloning and expression of the Bacteroides fragilis YCH46 neuraminidase gene in Escherichia coli and Bacteroides uniformis. FEMS Microbiol Lett 121:153–158. doi:10.1111/j.1574-6968.1994.tb07092.x 7926664

[13] Cato EP, Johnson JL. 1976. Reinstatement of species rank for Bacteroides fragilis, B. ovatus, B. distasonis, B. thetaiotaomicron, and B. vulgatus: designation of neotype strains for Bacteroides fragilis (Veillon and Zuber) Castellani and Chalmers and Bacteroides thetaiotaomicron (Distaso) Castellani and Chalmers. Int J Syst Bacteriol 26:230–237. doi:10.1099/00207713-26-2-230

[14] Sakamoto M, Benno Y. 2006. Reclassification of Bacteroides distasonis, Bacteroides goldsteinii and Bacteroides merdae as Parabacteroides distasonis gen. nov. Int J Syst Evol Microbiol 56:1599–1605. doi:10.1099/ijs.0.64192-0 16825636

[15] Oren A, Garrity GM. 2021. Valid publication of the names of forty-two phyla of prokaryotes. Int J Syst Evol Microbiol 71:10. doi:10.1099/ijsem.0.005056 34694987

[16] Hitch TCA, Bisdorf K, Afrizal A, Riedel T, Overmann J, Strowig T, Clavel T. 2022. A taxonomic note on the genus Prevotella: description of four novel genera and emended description of the genera Hallella and Xylanibacter. Syst Appl Microbiol 45:126354. doi:10.1016/j.syapm.2022.126354 36067550

[17] Blanco-Míguez A, Gálvez EJC, Pasolli E, De Filippis F, Amend L, Huang KD, Manghi P, Lesker T-R, Riedel T, Cova L, Punčochář M, Thomas AM, Valles-Colomer M, Schober I, Hitch TCA, Clavel T, Berry SE, Davies R, Wolf J, Spector TD, Overmann J, Tett A, Ercolini D, Segata N, Strowig T. 2023. Extension of the Segatella copri complex to 13 species with distinct large extrachromosomal elements and associations with host conditions. Cell Host Microbe 31:1804–1819. doi:10.1016/j.chom.2023.09.013 37883976 PMC10635906

[18] Gutacker M., Valsangiacomo C, Piffaretti JC. 2000. Identification of two genetic groups in Bacteroides fragilis by multilocus enzyme electrophoresis: distribution of antibiotic resistance (cfiA, cepA) and enterotoxin (bft) encoding genes. Microbiology (Reading) 146 (Pt 5):1241–1254. doi:10.1099/00221287-146-5-1241 10832652

[19] Gutacker Michaela, Valsangiacomo C, Bernasconi MV, Piffaretti J-C. 2002. RecA and glnA sequences separate the Bacteroides fragilis population into two genetic divisions associated with the antibiotic resistance genotypes cepA and cfiA. J Med Microbiol 51:123–130. doi:10.1099/0022-1317-51-2-123 11863263

[20] Ko KS, Kuwahara T, Haehwa L, Yoon Y-J, Kim B-J, Lee K-H, Ohnishi Y, Kook Y-H. 2007. RNA polymerase beta-subunit gene (rpoB) sequence analysis for the identification of Bacteroides spp. Clin Microbiol Infect 13:48–54. doi:10.1111/j.1469-0691.2006.01553.x 17184287

[21] Nagy E, Becker S, Sóki J, Urbán E, Kostrzewa M. 2011. Differentiation of division I (cfiA-negative) and division II (cfiA-positive) Bacteroides fragilis strains by matrix-assisted laser desorption/ionization time-of-flight mass spectrometry. J Med Microbiol 60:1584–1590. doi:10.1099/jmm.0.031336-0 21680764

[22] Bjerke GA, Wilson R, Storrø O, Øyen T, Johnsen R, Rudi K. 2011. Mother-to-child transmission of and multiple-strain colonization by Bacteroides fragilis in a cohort of mothers and their children. Appl Environ Microbiol 77:8318–8324. doi:10.1128/AEM.05293-11 21965394 PMC3233040

[23] Oles RE, Carrillo Terrazas M, Loomis LR, Hsu C-Y, Tribelhorn C, Belda-Ferre P, Ea AC, Bryant M, Young JA, Carrow HC, Sandborn WJ, Dulai PS, Sivagnanam M, Pride D, Knight R, Chu H. 2024. Pangenome comparison of Bacteroides fragilis genomospecies unveils genetic diversity and ecological insights. mSystems 9:e0051624. doi:10.1128/msystems.00516-24 38934546 PMC11265264

[24] Muto Y, Tanaka K. 2024. Comparative evolutionary genomics reveals genetic diversity and differentiation in Bacteroides fragilis. Genes (Basel) 15:12. doi:10.3390/genes15121519 PMC11675351 39766787

[25] English J, Newberry F, Hoyles L, Patrick S, Stewart L. 2023. Genomic analyses of Bacteroides fragilis: subdivisions I and II represent distinct species. J Med Microbiol 72:11. doi:10.1099/jmm.0.001768 37910167

[26] Rautio M, et al.. 2003. Reclassification of Bacteroides putredinis (Weinberg et al., 1937) in a new genus Alistipes gen. nov., as Alistipes putredinis comb. nov., and description of Alistipes finegoldii sp. nov., from human sources.. Syst Appl Microbiol 26:182–188. doi:10.1078/072320203322346029 12866844

[27] Sakamoto M, Lan PTN, Benno Y. 2007. Barnesiella viscericola gen. nov., sp. nov., a novel member of the family Porphyromonadaceae isolated from chicken caecum. Int J Syst Evol Microbiol 57:342–346. doi:10.1099/ijs.0.64709-0 17267976

[28] Hardham JM, King KW, Dreier K, Wong J, Strietzel C, Eversole RR, Sfintescu C, Evans RT. 2008. Transfer of Bacteroides splanchnicus to Odoribacter gen. nov. as Odoribacter splanchnicus comb. nov., and description of Odoribacter denticanis sp. nov., isolated from the crevicular spaces of canine periodontitis patients. Int J Syst Evol Microbiol 58:103–109. doi:10.1099/ijs.0.63458-0 18175692

[29] Sakamoto M, et al.. 2009. Butyricimonas synergistica gen. nov., sp. nov. and Butyricimonas virosa sp. nov., butyric acid-producing bacteria in the family “Porphyromonadaceae” isolated from rat faeces. Int J Syst Evol Microbiol 59:1748–1753. doi:10.1099/ijs.0.007674-0 19542124

[30] Salyers AA, Vercellotti JR, West SE, Wilkins TD. 1977. Fermentation of mucin and plant polysaccharides by strains of Bacteroides from the human colon. Appl Environ Microbiol 33:319–322. doi:10.1128/aem.33.2.319-322.1977 848954 PMC170684

[31] Salyers AA, Pajeau M. 1989. Competitiveness of different polysaccharide utilization mutants of Bacteroides thetaiotaomicron in the intestinal tracts of germfree mice. Appl Environ Microbiol 55:2572–2578. doi:10.1128/aem.55.10.2572-2578.1989 2557798 PMC203124

[32] Bry L, Falk PG, Midtvedt T, Gordon JI. 1996. A model of host-microbial interactions in an open mammalian ecosystem. Science 273:1380–1383. doi:10.1126/science.273.5280.1380 8703071

[33] Xu J, Bjursell MK, Himrod J, Deng S, Carmichael LK, Chiang HC, Hooper LV, Gordon JI. 2003. A genomic view of the human-Bacteroides thetaiotaomicron symbiosis. Science 299:2074–2076. doi:10.1126/science.1080029 12663928

[34] Koropatkin NM, Martens EC, Gordon JI, Smith TJ. 2008. Starch catabolism by a prominent human gut symbiont is directed by the recognition of amylose helices. Structure 16:1105–1115. doi:10.1016/j.str.2008.03.017 18611383 PMC2563962

[35] Goodman AL, McNulty NP, Zhao Y, Leip D, Mitra RD, Lozupone CA, Knight R, Gordon JI. 2009. Identifying genetic determinants needed to establish a human gut symbiont in its habitat. Cell Host Microbe 6:279–289. doi:10.1016/j.chom.2009.08.003 19748469 PMC2895552

[36] Hooper LV, Wong MH, Thelin A, Hansson L, Falk PG, Gordon JI. 2001. Molecular analysis of commensal host-microbial relationships in the intestine. Science 291:881–884. doi:10.1126/science.291.5505.881 11157169

[37] Bäckhed F, Ding H, Wang T, Hooper LV, Koh GY, Nagy A, Semenkovich CF, Gordon JI. 2004. The gut microbiota as an environmental factor that regulates fat storage. Proc Natl Acad Sci USA 101:15718–15723. doi:10.1073/pnas.0407076101 15505215 PMC524219

[38] Ley RE, Bäckhed F, Turnbaugh P, Lozupone CA, Knight RD, Gordon JI. 2005. Obesity alters gut microbial ecology. Proc Natl Acad Sci USA 102:11070–11075. doi:10.1073/pnas.0504978102 16033867 PMC1176910

[39] Turnbaugh PJ, Ley RE, Mahowald MA, Magrini V, Mardis ER, Gordon JI. 2006. An obesity-associated gut microbiome with increased capacity for energy harvest. Nature 444:1027–1031. doi:10.1038/nature05414 17183312

[40] Ley RE, Turnbaugh PJ, Klein S, Gordon JI. 2006. Microbial ecology: human gut microbes associated with obesity. Nature 444:1022–1023. doi:10.1038/4441022a 17183309

[41] Suau A, Bonnet R, Sutren M, Godon JJ, Gibson GR, Collins MD, Doré J. 1999. Direct analysis of genes encoding 16S rRNA from complex communities reveals many novel molecular species within the human gut. Appl Environ Microbiol 65:4799–4807. doi:10.1128/AEM.65.11.4799-4807.1999 10543789 PMC91647

[42] Eckburg PB, Bik EM, Bernstein CN, Purdom E, Dethlefsen L, Sargent M, Gill SR, Nelson KE, Relman DA. 2005. Diversity of the human intestinal microbial flora. Science 308:1635–1638. doi:10.1126/science.1110591 15831718 PMC1395357

[43] Bäckhed F, Ley RE, Sonnenburg JL, Peterson DA, Gordon JI. 2005. Host-bacterial mutualism in the human intestine. Science 307:1915–1920. doi:10.1126/science.1104816 15790844

[44] Kuwahara T, Yamashita A, Hirakawa H, Nakayama H, Toh H, Okada N, Kuhara S, Hattori M, Hayashi T, Ohnishi Y. 2004. Genomic analysis of Bacteroides fragilis reveals extensive DNA inversions regulating cell surface adaptation. Proc Natl Acad Sci USA 101:14919–14924. doi:10.1073/pnas.0404172101 15466707 PMC522005

[45] Cerdeño-Tárraga AM, Patrick S, Crossman LC, Blakely G, Abratt V, Lennard N, Poxton I, Duerden B, Harris B, Quail MA, Barron A, Clark L, Corton C, Doggett J, Holden MTG, Larke N, Line A, Lord A, Norbertczak H, Ormond D, Price C, Rabbinowitsch E, Woodward J, Barrell B, Parkhill J. 2005. Extensive DNA inversions in the B. fragilis genome control variable gene expression. Science 307:1463–1465. doi:10.1126/science.1107008 15746427

[46] Xu J, Mahowald MA, Ley RE, Lozupone CA, Hamady M, Martens EC, Henrissat B, Coutinho PM, Minx P, Latreille P, Cordum H, Van Brunt A, Kim K, Fulton RS, Fulton LA, Clifton SW, Wilson RK, Knight RD, Gordon JI. 2007. Evolution of symbiotic bacteria in the distal human intestine. PLoS Biol 5:e156. doi:10.1371/journal.pbio.0050156 17579514 PMC1892571

[47] Garrod LP. 1955. Sensitivity of four species of bacteroides to antibiotics. Br Med J 2:1529–1531. doi:10.1136/bmj.2.4955.1529 13269908 PMC1981436

[48] Stevens WC, Harrison AP Jr. 1958. In vitro antibiotic sensitivities of Bacteroides and similar forms. Antibiot Chemother (Northfield) 8:192–194. 24544722

[49] Finegold SM, Hewitt WL. 1955. Antibiotic sensitivity pattern of Bacteroides species. Antibiot Annu 3:794–801. 13355365

[50] Snydman DR, McDermott L, Cuchural GJ Jr, Hecht DW, Iannini PB, Harrell LJ, Jenkins SG, O’Keefe JP, Pierson CL, Rihs JD, Yu VL, Finegold SM, Gorbach SL. 1996. Analysis of trends in antimicrobial resistance patterns among clinical isolates of Bacteroides fragilis group species from 1990 to 1994. Clin Infect Dis 23 Suppl 1:S54–65. doi:10.1093/clinids/23.supplement_1.s54 8953108

[51] Snydman DR, Jacobus NV, McDermott LA, Goldstein EJC, Harrell L, Jenkins SG, Newton D, Patel R, Hecht DW. 2017. Trends in antimicrobial resistance among Bacteroides species and Parabacteroides species in the United States from 2010-2012 with comparison to 2008-2009. Anaerobe 43:21–26. doi:10.1016/j.anaerobe.2016.11.003 27867083

[52] Wu ZC, Feng HX, Wu L, Zhang M, Gu Z. 2025. Antimicrobial resistance pattern of the Bacteroides fragilis group strains isolated at a teaching hospital in China. Acta Microbiol Immunol Hung 72:59–67. doi:10.1556/030.2025.02400 39907717

[53] Bodner SJ, Koenig MG, Goodman JS. 1970. Bacteremic Bacteroides infections. Ann Intern Med 73:537–544. doi:10.7326/0003-4819-73-4-537 5506005

[54] Bodner SJ, Koenig MG, Treanor LL, Goodman JS. 1972. Antibiotic susceptibility testing of Bacteroides. Antimicrob Agents Chemother 2:57–60. doi:10.1128/AAC.2.2.57 4670488 PMC444267

[55] Sutter VL, Kwok YY, Finegold SM. 1972. Standardized antimicrobial disc susceptibility testing of anaerobic bacteria. I. Susceptibility of Bacteroides fragilis to tetracycline. Appl Microbiol 23:268–275. doi:10.1128/am.23.2.268-275.1972 5017675 PMC380328

[56] Mancini C, Behme RJ. 1977. Transfer of multiple antibiotic resistance from Bacteroides fragilis to Escherichia coli. J Infect Dis 136:597–600. doi:10.1093/infdis/136.4.597 333042

[57] Tally FP, Snydman DR, Gorbach SL, Malamy MH. 1979. Plasmid-mediated, transferable resistance to clindamycin and erythromycin in Bacteroides fragilis. J Infect Dis 139:83–88. doi:10.1093/infdis/139.1.83 438530

[58] Tally FP, Malamy MH. 1982. Mechanism of antimicrobial resistance and resistance transfer in anaerobic bacteria. Scand J Infect Dis Suppl 35:37–44. 6300995

[59] Welch RA, Jones KR, Macrina FL. 1979. Transferable lincosamide-macrolide resistance in Bacteroides. Plasmid 2:261–268. doi:10.1016/0147-619x(79)90044-1 451051

[60] Smith CJ, Rogers MB, McKee ML. 1992. Heterologous gene expression in Bacteroides fragilis. Plasmid 27:141–154. doi:10.1016/0147-619x(92)90014-2 1615064

[61] Shoemaker NB, Smith MD, Guild WR. 1979. Organization and transfer of heterologous chloramphenicol and tetracycline resistance genes in pneumococcus. J Bacteriol 139:432–441. doi:10.1128/jb.139.2.432-441.1979 37238 PMC216887

[62] Shoemaker NB, Smith MD, Guild WR. 1980. DNase-resistant transfer of chromosomal cat and tet insertions by filter mating in Pneumococcus. Plasmid 3:80–87. doi:10.1016/s0147-619x(80)90036-0 6278526

[63] Gawron-Burke C, Clewell DB. 1982. A transposon in Streptococcus faecalis with fertility properties. Nature 300:281–284. doi:10.1038/300281a0 6292725

[64] Hochhut B, Waldor MK. 1999. Site-specific integration of the conjugal Vibrio cholerae SXT element into prfC. Mol Microbiol 32:99–110. doi:10.1046/j.1365-2958.1999.01330.x 10216863

[65] Hentschel U, Hacker J. 2001. Pathogenicity islands: the tip of the iceberg. Microbes Infect 3:545–548. doi:10.1016/S1286-4579(01)01410-1 11418328

[66] Burrus V, Pavlovic G, Decaris B, Guédon G. 2002. Conjugative transposons: the tip of the iceberg. Mol Microbiol 46:601–610. doi:10.1046/j.1365-2958.2002.03191.x 12410819

[67] Shoemaker NB, Salyers AA. 1990. A cryptic 65-kilobase-pair transposonlike element isolated from Bacteroides uniformis has homology with Bacteroides conjugal tetracycline resistance elements. J Bacteriol 172:1694–1702. doi:10.1128/jb.172.4.1694-1702.1990 2156799 PMC208658

[68] Shoemaker NB, Barber RD, Salyers AA. 1989. Cloning and characterization of a Bacteroides conjugal tetracycline-erythromycin resistance element by using a shuttle cosmid vector. J Bacteriol 171:1294–1302. doi:10.1128/jb.171.3.1294-1302.1989 2646276 PMC209744

[69] Bedzyk LA, Shoemaker NB, Young KE, Salyers AA. 1992. Insertion and excision of Bacteroides conjugative chromosomal elements. J Bacteriol 174:166–172. doi:10.1128/jb.174.1.166-172.1992 1309516 PMC205691

[70] Wozniak RAF, Waldor MK. 2010. Integrative and conjugative elements: mosaic mobile genetic elements enabling dynamic lateral gene flow. Nat Rev Microbiol 8:552–563. doi:10.1038/nrmicro2382 20601965

[71] Coyne MJ, Zitomersky NL, McGuire AM, Earl AM, Comstock LE. 2014. Evidence of extensive DNA transfer between Bacteroidales species within the human gut. mBio 5:e01305–14. doi:10.1128/mBio.01305-14 24939888 PMC4073490

[72] García-Bayona L, Coyne MJ, Comstock LE. 2021. Mobile Type VI secretion system loci of the gut Bacteroidales display extensive intra-ecosystem transfer, multi-species spread and geographical clustering. PLoS Genet 17:e1009541. doi:10.1371/journal.pgen.1009541 33901198 PMC8102008

[73] Brito IL, Yilmaz S, Huang K, Xu L, Jupiter SD, Jenkins AP, Naisilisili W, Tamminen M, Smillie CS, Wortman JR, Birren BW, Xavier RJ, Blainey PC, Singh AK, Gevers D, Alm EJ. 2016. Mobile genes in the human microbiome are structured from global to individual scales. Nature 535:435–439. doi:10.1038/nature18927 27409808 PMC4983458

[74] Zhang ZJ, Cole CG, Coyne MJ, Lin H, Dylla N, Smith RC, Pappas TE, Townson SA, Laliwala N, Waligurski E, Ramaswamy R, Woodson C, Burgo V, Little JC, Moran D, Rose A, McMillin M, McSpadden E, Sundararajan A, Sidebottom AM, Pamer EG, Comstock LE. 2024. Comprehensive analyses of a large human gut Bacteroidales culture collection reveal species- and strain-level diversity and evolution. Cell Host Microbe 32:1853–1867. doi:10.1016/j.chom.2024.08.016 39293438 PMC11466702

[75] Coyne M.J., Roelofs KG, Comstock LE. 2016. Type VI secretion systems of human gut Bacteroidales segregate into three genetic architectures, two of which are contained on mobile genetic elements. BMC Genomics 17:58. doi:10.1186/s12864-016-2377-z 26768901 PMC4714493

[76] Coyne Michael J, Béchon N, Matano LM, McEneany VL, Chatzidaki-Livanis M, Comstock LE. 2019. A family of anti-Bacteroidales peptide toxins wide-spread in the human gut microbiota. Nat Commun 10:3460. doi:10.1038/s41467-019-11494-1 31371723 PMC6671954

[77] Ross BD, Verster AJ, Radey MC, Schmidtke DT, Pope CE, Hoffman LR, Hajjar AM, Peterson SB, Borenstein E, Mougous JD. 2019. Human gut bacteria contain acquired interbacterial defence systems. Nature 575:224–228. doi:10.1038/s41586-019-1708-z 31666699 PMC6938237

[78] Pudlo NA, Pereira GV, Parnami J, Cid M, Markert S, Tingley JP, Unfried F, Ali A, Varghese NJ, Kim KS, Campbell A, Urs K, Xiao Y, Adams R, Martin D, Bolam DN, Becher D, Eloe-Fadrosh EA, Schmidt TM, Abbott DW, Schweder T, Hehemann JH, Martens EC. 2022. Diverse events have transferred genes for edible seaweed digestion from marine to human gut bacteria. Cell Host Microbe 30:314–328. doi:10.1016/j.chom.2022.02.001 35240043 PMC9096808

[79] Frye KA, Piamthai V, Hsiao A, Degnan PH. 2021. Mobilization of vitamin B(12) transporters alters competitive dynamics in a human gut microbe. Cell Rep 37:110164. doi:10.1016/j.celrep.2021.110164 34965410 PMC8759732

[80] Waters JL, Salyers AA. 2013. Regulation of CTnDOT conjugative transfer is a complex and highly coordinated series of events. mBio 4:e00569-13. doi:10.1128/mBio.00569-13 PMC3809561 24169574

[81] Salyers AA, Palmer JK, Wilkins TD. 1978. Degradation of polysaccharides by intestinal bacterial enzymes. Am J Clin Nutr 31:S128–S130. doi:10.1093/ajcn/31.10.S128 707363

[82] Salyers AA, Harris CJ, Wilkins TD. 1978. Breakdown of psyllium hydrocolloid by strains of Bacterioides ovatus from the human intestinal tract. Can J Microbiol 24:336–338. doi:10.1139/m78-057 647481

[83] Betian HG, Linehan BA, Bryant MP, Holdeman LV. 1977. Isolation of a cellulotytic Bacteroides sp. from human feces. Appl Environ Microbiol 33:1009–1010. doi:10.1128/aem.33.4.1009-1010.1977 869523 PMC170812

[84] Anderson KL, Salyers AA. 1989 a. Biochemical evidence that starch breakdown by Bacteroides thetaiotaomicron involves outer membrane starch-binding sites and periplasmic starch-degrading enzymes. J Bacteriol 171:3192–3198. doi:10.1128/jb.171.6.3192-3198.1989 2722747 PMC210036

[85] Anderson KL 1989 b. Genetic evidence that outer membrane binding of starch is required for starch utilization by Bacteroides thetaiotaomicron. J Bacteriol 171:3199–3204. doi:10.1128/jb.171.6.3199-3204.1989 2722748 PMC210037

[86] Tancula E, Feldhaus MJ, Bedzyk LA, Salyers AA. 1992. Location and characterization of genes involved in binding of starch to the surface of Bacteroides thetaiotaomicron. J Bacteriol 174:5609–5616. doi:10.1128/jb.174.17.5609-5616.1992 1512196 PMC206506

[87] Reeves AR, et al.. 1996. A Bacteroides thetaiotaomicron outer membrane protein that is essential for utilization of maltooligosaccharides and starch. J Bacteriol 178:823–830. 8550519 10.1128/jb.178.3.823-830.1996 PMC177731

[88] D’Elia JN, Salyers AA. 1996. Contribution of a neopullulanase, a pullulanase, and an alpha-glucosidase to growth of Bacteroides thetaiotaomicron on starch. J Bacteriol 178:7173–7179. doi:10.1128/jb.178.24.7173-7179.1996 8955399 PMC178630

[89] Reeves AR, Wang GR, Salyers AA. 1997. Characterization of four outer membrane proteins that play a role in utilization of starch by Bacteroides thetaiotaomicron. J Bacteriol 179:643–649. doi:10.1128/jb.179.3.643-649.1997 9006015 PMC178742

[90] Shipman JA, Cho KH, Siegel HA, Salyers AA. 1999. Physiological characterization of SusG, an outer membrane protein essential for starch utilization by Bacteroides thetaiotaomicron. J Bacteriol 181:7206–7211. doi:10.1128/JB.181.23.7206-7211.1999 10572122 PMC103681

[91] Cho KH, Salyers AA. 2001. Biochemical analysis of interactions between outer membrane proteins that contribute to starch utilization by Bacteroides thetaiotaomicron. J Bacteriol 183:7224–7230. doi:10.1128/JB.183.24.7224-7230.2001 11717282 PMC95572

[92] D’Elia JN, Salyers AA. 1996. Effect of regulatory protein levels on utilization of starch by Bacteroides thetaiotaomicron. J Bacteriol 178:7180–7186. doi:10.1128/jb.178.24.7180-7186.1996 8955400 PMC178631

[93] Cho KH, Cho D, Wang GR, Salyers AA. 2001. New regulatory gene that contributes to control of Bacteroides thetaiotaomicron starch utilization genes. J Bacteriol 183:7198–7205. doi:10.1128/JB.183.24.7198-7205.2001 11717279 PMC95569

[94] Bjursell MK, Martens EC, Gordon JI. 2006. Functional genomic and metabolic studies of the adaptations of a prominent adult human gut symbiont, Bacteroides thetaiotaomicron, to the suckling period. J Biol Chem 281:36269–36279. doi:10.1074/jbc.M606509200 16968696

[95] Sonnenburg JL, Xu J, Leip DD, Chen C-H, Westover BP, Weatherford J, Buhler JD, Gordon JI. 2005. Glycan foraging in vivo by an intestine-adapted bacterial symbiont. Science 307:1955–1959. doi:10.1126/science.1109051 15790854

[96] Martens EC, Chiang HC, Gordon JI. 2008. Mucosal glycan foraging enhances fitness and transmission of a saccharolytic human gut bacterial symbiont. Cell Host Microbe 4:447–457. doi:10.1016/j.chom.2008.09.007 18996345 PMC2605320

[97] Martens EC, Lowe EC, Chiang H, Pudlo NA, Wu M, McNulty NP, Abbott DW, Henrissat B, Gilbert HJ, Bolam DN, Gordon JI. 2011. Recognition and degradation of plant cell wall polysaccharides by two human gut symbionts. PLoS Biol 9:e1001221. doi:10.1371/journal.pbio.1001221 22205877 PMC3243724

[98] Rogers TE, Pudlo NA, Koropatkin NM, Bell JSK, Moya Balasch M, Jasker K, Martens EC. 2013. Dynamic responses of Bacteroides thetaiotaomicron during growth on glycan mixtures. Mol Microbiol 88:876–890. doi:10.1111/mmi.12228 23646867 PMC3700664

[99] Glenwright AJ, Pothula KR, Bhamidimarri SP, Chorev DS, Baslé A, Firbank SJ, Zheng H, Robinson CV, Winterhalter M, Kleinekathöfer U, Bolam DN, van den Berg B. 2017. Structural basis for nutrient acquisition by dominant members of the human gut microbiota. Nature 541:407–411. doi:10.1038/nature20828 28077872 PMC5497811

[100] Gray DA, White JBR, Oluwole AO, Rath P, Glenwright AJ, Mazur A, Zahn M, Baslé A, Morland C, Evans SL, Cartmell A, Robinson CV, Hiller S, Ranson NA, Bolam DN, van den Berg B. 2021. Insights into SusCD-mediated glycan import by a prominent gut symbiont. Nat Commun 12:44. doi:10.1038/s41467-020-20285-y 33398001 PMC7782687

[101] Pudlo NA, Urs K, Kumar SS, German JB, Mills DA, Martens EC. 2015. Symbiotic human gut bacteria with variable metabolic priorities for host mucosal glycans. mBio 6:e01282-15. doi:10.1128/mBio.01282-15 26556271 PMC4659458

[102] Pudlo NA, Urs K, Crawford R, Pirani A, Atherly T, Jimenez R, Terrapon N, Henrissat B, Peterson D, Ziemer C, Snitkin E, Martens EC. 2022. Phenotypic and genomic diversification in complex carbohydrate-degrading human gut bacteria. mSystems 7:e0094721. doi:10.1128/msystems.00947-21 35166563 PMC8845570

[103] Gellman RH, Olm MR, Terrapon N, Enam F, Higginbottom SK, Sonnenburg JL, Sonnenburg ED. 2023. Hadza Prevotella require diet-derived microbiota-accessible carbohydrates to persist in mice. Cell Rep 42:11. doi:10.1016/j.celrep.2023.113233 PMC10954246 38510311

[104] Onderdonk AB, Kasper DL, Cisneros RL, Bartlett JG. 1977. The capsular polysaccharide of Bacteroides fragilis as a virulence factor: comparison of the pathogenic potential of encapsulated and unencapsulated Strains. Journal of Infectious Diseases 136:82–89. doi:10.1093/infdis/136.1.82 886206

[105] Pantosti A, Tzianabos AO, Onderdonk AB, Kasper DL. 1991. Immunochemical characterization of two surface polysaccharides of Bacteroides fragilis. Infect Immun 59:2075–2082. doi:10.1128/iai.59.6.2075-2082.1991 2037368 PMC257968

[106] Tzianabos AO, Onderdonk AB, Rosner B, Cisneros RL, Kasper DL. 1993. Structural features of polysaccharides that induce intra-abdominal abscesses. Science 262:416–419. doi:10.1126/science.8211161 8211161

[107] Krinos CM, Coyne MJ, Weinacht KG, Tzianabos AO, Kasper DL, Comstock LE. 2001. Extensive surface diversity of a commensal microorganism by multiple DNA inversions. Nature 414:555–558. doi:10.1038/35107092 11734857

[108] Coyne MJ, Weinacht KG, Krinos CM, Comstock LE. 2003. Mpi recombinase globally modulates the surface architecture of a human commensal bacterium. Proc Natl Acad Sci USA 100:10446–10451. doi:10.1073/pnas.1832655100 12915735 PMC193581

[109] Chatzidaki-Livanis M, Coyne MJ, Comstock LE. 2009. A family of transcriptional antitermination factors necessary for synthesis of the capsular polysaccharides of Bacteroides fragilis. J Bacteriol 191:7288–7295. doi:10.1128/JB.00500-09 19801412 PMC2786560

[110] Chatzidaki-Livanis M, Weinacht KG, Comstock LE. 2010. Trans locus inhibitors limit concomitant polysaccharide synthesis in the human gut symbiont Bacteroides fragilis. Proc Natl Acad Sci USA 107:11976–11980. doi:10.1073/pnas.1005039107 20547868 PMC2900635

[111] Saba J, Flores K, Marshall B, Engstrom MD, Peng Y, Garje AS, Comstock LE, Landick R. 2024. Bacteroides expand the functional versatility of a conserved transcription factor and transcribed DNA to program capsule diversity. Nat Commun 15:10862. doi:10.1038/s41467-024-55215-9 39738018 PMC11685472

[112] Coyne MJ, Comstock LE. 2008. Niche-specific features of the intestinal bacteroidales. J Bacteriol 190:736–742. doi:10.1128/JB.01559-07 17993536 PMC2223690

[113] Coyne MJ, Reinap B, Lee MM, Comstock LE. 2005. Human symbionts use a host-like pathway for surface fucosylation. Science 307:1778–1781. doi:10.1126/science.1106469 15774760

[114] Fletcher CM, Coyne MJ, Villa OF, Chatzidaki-Livanis M, Comstock LE. 2009. A general O-glycosylation system important to the physiology of a major human intestinal symbiont. Cell 137:321–331. doi:10.1016/j.cell.2009.02.041 19379697 PMC2772059

[115] Hoffmanns L, Svedberg D, Mateus A. 2025. Protein O-glycosylation in the Bacteroidota phylum. FEBS Open Bio. doi:10.1002/2211-5463.70041 PMC12871550 40231347

[116] Weinacht KG, Roche H, Krinos CM, Coyne MJ, Parkhill J, Comstock LE. 2004. Tyrosine site-specific recombinases mediate DNA inversions affecting the expression of outer surface proteins of Bacteroides fragilis. Mol Microbiol 53:1319–1330. doi:10.1111/j.1365-2958.2004.04219.x 15387812

[117] Roche-Hakansson H, Chatzidaki-Livanis M, Coyne MJ, Comstock LE. 2007. Bacteroides fragilis synthesizes a DNA invertase affecting both a local and a distant region. J Bacteriol 189:2119–2124. doi:10.1128/JB.01362-06 17189372 PMC1855777

[118] Nakayama-Imaohji H, Hirota K, Yamasaki H, Yoneda S, Nariya H, Suzuki M, Secher T, Miyake Y, Oswald E, Hayashi T, Kuwahara T. 2016. DNA Inversion Regulates Outer Membrane Vesicle Production in Bacteroides fragilis. PLoS One 11:e0148887. doi:10.1371/journal.pone.0148887 26859882 PMC4747536

[119] Chatzidaki-Livanis M, Coyne MJ, Roche-Hakansson H, Comstock LE. 2008. Expression of a uniquely regulated extracellular polysaccharide confers a large-capsule phenotype to Bacteroides fragilis. J Bacteriol 190:1020–1026. doi:10.1128/JB.01519-07 18039760 PMC2223589

[120] Ben-Assa N, Coyne MJ, Fomenkov A, Livny J, Robins WP, Muniesa M, Carey V, Carasso S, Gefen T, Jofre J, Roberts RJ, Comstock LE, Geva-Zatorsky N. 2020. Analysis of a phase-variable restriction modification system of the human gut symbiont Bacteroides fragilis. Nucleic Acids Res 48:11040–11053. doi:10.1093/nar/gkaa824 33045731 PMC7641763

[121] Huang X, Wang J, Li J, Liu Y, Liu X, Li Z, Kurniyati K, Deng Y, Wang G, Ralph JD, De Ste Croix M, Escobar-Gonzalez S, Roberts RJ, Veening J-W, Lan X, Oggioni MR, Li C, Zhang J-R. 2020. Prevalence of phase variable epigenetic invertons among host-associated bacteria. Nucleic Acids Res 48:11468–11485. doi:10.1093/nar/gkaa907 33119758 PMC7672463

[122] Nakayama-Imaohji H, Hirakawa H, Ichimura M, Wakimoto S, Kuhara S, Hayashi T, Kuwahara T. 2009. Identification of the site-specific DNA invertase responsible for the phase variation of SusC/SusD family outer membrane proteins in Bacteroides fragilis. J Bacteriol 191:6003–6011. doi:10.1128/JB.00687-09 19648246 PMC2747901

[123] Chanin RB, West PT, Wirbel J, Gill MO, Green GZM, Park RM, Enright N, Miklos AM, Hickey AS, Brooks EF, Lum KK, Cristea IM, Bhatt AS. 2024. Intragenic DNA inversions expand bacterial coding capacity. Nature 634:234–242. doi:10.1038/s41586-024-07970-4 39322669

[124] Maskell JP. 1994. Electrophoretic analysis of the lipopolysaccharides of Bacteroides spp. Antonie Van Leeuwenhoek 65:155–161. doi:10.1007/BF00871756 7979320

[125] Di Lorenzo F, Pither MD, Martufi M, Scarinci I, Guzmán-Caldentey J, Łakomiec E, Jachymek W, Bruijns SCM, Santamaría SM, Frick J-S, van Kooyk Y, Chiodo F, Silipo A, Bernardini ML, Molinaro A. 2020. Pairing Bacteroides vulgatus LPS structure with its immunomodulatory effects on human cellular models. ACS Cent Sci 6:1602–1616. doi:10.1021/acscentsci.0c00791 32999936 PMC7517413

[126] Pither MD, Illiano A, Pagliuca C, Jacobson A, Mantova G, Stornaiuolo A, Colicchio R, Vitiello M, Pinto G, Silipo A, Fischbach MA, Salvatore P, Amoresano A, Molinaro A, Di Lorenzo F. 2022. Bacteroides thetaiotaomicron rough-type lipopolysaccharide: the chemical structure and the immunological activity. Carbohydr Polym 297:120040. doi:10.1016/j.carbpol.2022.120040 36184180

[127] Tiemblo Martín M, Coccimiglio M, Andretta E, De Simone Carone L, Bell A, Gerpe-Amor T, Di Carluccio C, Molinaro A, van Kooyk Y, Juge N, Chiodo F, Di Lorenzo F, Silipo A. 2025. The human gut Bacteroides eggerthii expresses a new galactofuranose-containing lipooligosaccharide with weak immunostimulatory properties. Carbohydr Polym 348:122833. doi:10.1016/j.carbpol.2024.122833 39562107

[128] Patrick S, Houston S, Thacker Z, Blakely GW. 2009. Mutational analysis of genes implicated in LPS and capsular polysaccharide biosynthesis in the opportunistic pathogen Bacteroides fragilis. Microbiology (Reading) 155:1039–1049. doi:10.1099/mic.0.025361-0 19332806

[129] Peterson DA, Planer JD, Guruge JL, Xue L, Downey-Virgin W, Goodman AL, Seedorf H, Gordon JI. 2015. Characterizing the interactions between a naturally primed immunoglobulin A and its conserved Bacteroides thetaiotaomicron species-specific epitope in gnotobiotic mice. J Biol Chem 290:12630–12649. doi:10.1074/jbc.M114.633800 25795776 PMC4432283

[130] Roelofs KG, Coyne MJ, Gentyala RR, Chatzidaki-Livanis M, Comstock LE. 2016. Bacteroidales secreted antimicrobial proteins target surface molecules necessary for gut colonization and mediate competition in vivo. mBio 7:e01055-16. doi:10.1128/mBio.01055-16 27555309 PMC4999547

[131] Jacobson AN, Choudhury BP, Fischbach MA. 2018. The biosynthesis of Lipooligosaccharide from Bacteroides thetaiotaomicron mBio 9:e02289-17. doi:10.1128/mBio.02289-17 29535205 PMC5850320

[132] McEneany VL, Coyne MJ, Chatzidaki-Livanis M, Comstock LE. 2018. Acquisition of MACPF domain-encoding genes is the main contributor to LPS glycan diversity in gut Bacteroides species. ISME J 12:2919–2928. doi:10.1038/s41396-018-0244-4 30065309 PMC6246601

[133] Kasper DL. 1976. Chemical and biological characterization of the Lipopolysaccharide of Bacteroides fragilis Subspecies fragilis. J Infect Dis 134:59–66. doi:10.1093/infdis/134.1.59 939922

[134] Hofstad T, Sveen K, Dahlén G. 1977. Chemical composition, serological reactivity and endotoxicity of lipopolysaccharides extracted in different ways from Bacteroides fragilis, Bacteroides melaninogenicus and Bacteroides oralis. Acta Pathol Microbiol Scand B 85:262–270. doi:10.1111/j.1699-0463.1977.tb01972.x 19922

[135] Weintraub A, Zähringer U, Wollenweber HW, Seydel U, Rietschel ET. 1989. Structural characterization of the lipid A component of Bacteroides fragilis strain NCTC 9343 lipopolysaccharide. Eur J Biochem 183:425–431. doi:10.1111/j.1432-1033.1989.tb14945.x 2759091

[136] Lindberg AA, Weintraub A, Zähringer U, Rietschel ET. 1990. Structure-activity relationships in lipopolysaccharides of Bacteroides fragilis. Rev Infect Dis 12 Suppl 2:S133–41. doi:10.1093/clinids/12.supplement_2.s133 2406867

[137] Hashimoto M, Kirikae F, Dohi T, Adachi S, Kusumoto S, Suda Y, Fujita T, Naoki H, Kirikae T. 2002. Structural study on lipid A and the O-specific polysaccharide of the lipopolysaccharide from a clinical isolate of Bacteroides vulgatus from a patient with Crohn’s disease. Eur J Biochem 269:3715–3721. doi:10.1046/j.1432-1033.2002.03062.x 12153568

[138] Cullen TW, Schofield WB, Barry NA, Putnam EE, Rundell EA, Trent MS, Degnan PH, Booth CJ, Yu H, Goodman AL. 2015. Gut microbiota. Antimicrobial peptide resistance mediates resilience of prominent gut commensals during inflammation. Science 347:170–175. doi:10.1126/science.1260580 25574022 PMC4388331

[139] Inouye S, Wang S, Sekizawa J, Halegoua S, Inouye M. 1977. Amino acid sequence for the peptide extension on the prolipoprotein of the Escherichia coli outer membrane. Proc Natl Acad Sci USA 74:1004–1008. doi:10.1073/pnas.74.3.1004 322142 PMC430563

[140] Lauber F, Cornelis GR, Renzi F. 2016. Identification of a new lipoprotein export signal in gram-negative bacteria. mBio 7. doi:10.1128/mBio.01232-16 PMC5080379 27795390

[141] Choi VM, Herrou J, Hecht AL, Teoh WP, Turner JR, Crosson S, Bubeck Wardenburg J. 2016. Activation of Bacteroides fragilis toxin by a novel bacterial protease contributes to anaerobic sepsis in mice. Nat Med 22:563–567. doi:10.1038/nm.4077 27089515 PMC4860040

[142] Herrou J, Choi VM, Bubeck Wardenburg J, Crosson S. 2016. Activation mechanism of the Bacteroides fragilis Cysteine Peptidase, Fragipain. Biochemistry 55:4077–4084. doi:10.1021/acs.biochem.6b00546 27379832 PMC5053104

[143] Pierce JV, Fellows JD, Anderson DE, Bernstein HD. 2021. A clostripain-like protease plays a major role in generating the secretome of enterotoxigenic Bacteroides fragilis. Mol Microbiol 115:290–304. doi:10.1111/mmi.14616 32996200 PMC9358847

[144] Bao Y, Verdegaal AA, Anderson BW, Barry NA, He J, Gao X, Goodman AL. 2021. A common pathway for activation of host-targeting and bacteria-targeting toxins in human intestinal bacteria. mBio 12:e0065621. doi:10.1128/mBio.00656-21 34465018 PMC8406203

[145] Evans JC, McEneany VL, Coyne MJ, Caldwell EP, Sheahan ML, Von SS, Coyne EM, Tweten RK, Comstock LE. 2022. A proteolytically activated antimicrobial toxin encoded on a mobile plasmid of Bacteroidales induces a protective response. Nat Commun 13:4258. doi:10.1038/s41467-022-31925-w 35871068 PMC9308784

[146] Abrahamsen HL, Sanford TC, Collamore CE, Johnstone BA, Coyne MJ, García-Bayona L, Christie MP, Evans JC, Farrand AJ, Flores K, Morton CJ, Parker MW, Comstock LE, Tweten RK. 2024. Distant relatives of a eukaryotic cell-specific toxin family evolved a complement-like mechanism to kill bacteria. Nat Commun 15:5028. doi:10.1038/s41467-024-49103-5 38866748 PMC11169675

[147] Shen Y, Giardino Torchia ML, Lawson GW, Karp CL, Ashwell JD, Mazmanian SK. 2012. Outer membrane vesicles of a human commensal mediate immune regulation and disease protection. Cell Host Microbe 12:509–520. doi:10.1016/j.chom.2012.08.004 22999859 PMC3895402

[148] Stentz R, Osborne S, Horn N, Li AWH, Hautefort I, Bongaerts R, Rouyer M, Bailey P, Shears SB, Hemmings AM, Brearley CA, Carding SR. 2014. A bacterial homolog of a eukaryotic inositol phosphate signaling enzyme mediates cross-kingdom dialog in the mammalian gut. Cell Rep 6:646–656. doi:10.1016/j.celrep.2014.01.021 24529702 PMC3969271

[149] Hickey CA, Kuhn KA, Donermeyer DL, Porter NT, Jin C, Cameron EA, Jung H, Kaiko GE, Wegorzewska M, Malvin NP, Glowacki RWP, Hansson GC, Allen PM, Martens EC, Stappenbeck TS. 2015. Colitogenic Bacteroides thetaiotaomicron antigens access host immune cells in a sulfatase-dependent manner via outer membrane vesicles. Cell Host Microbe 17:672–680. doi:10.1016/j.chom.2015.04.002 25974305 PMC4432250

[150] Zakharzhevskaya NB, Tsvetkov VB, Vanyushkina AA, Varizhuk AM, Rakitina DV, Podgorsky VV, Vishnyakov IE, Kharlampieva DD, Manuvera VA, Lisitsyn FV, Gushina EA, Lazarev VN, Govorun VM. 2017. Interaction of Bacteroides fragilis toxin with outer membrane vesicles reveals new mechanism of its secretion and delivery. Front Cell Infect Microbiol 7:2. doi:10.3389/fcimb.2017.00002 28144586 PMC5240029

[151] Chu H, Khosravi A, Kusumawardhani IP, Kwon AHK, Vasconcelos AC, Cunha LD, Mayer AE, Shen Y, Wu W-L, Kambal A, Targan SR, Xavier RJ, Ernst PB, Green DR, McGovern DPB, Virgin HW, Mazmanian SK. 2016. Gene-microbiota interactions contribute to the pathogenesis of inflammatory bowel disease. Science 352:1116–1120. doi:10.1126/science.aad9948 27230380 PMC4996125

[152] Maerz JK, Steimle A, Lange A, Bender A, Fehrenbacher B, Frick J-S. 2018. Outer membrane vesicles blebbing contributes to B. vulgatus mpk-mediated immune response silencing. Gut Microbes 9:1–12. doi:10.1080/19490976.2017.1344810 PMC5914909 28686482

[153] Stentz R, Carvalho AL, Jones EJ, Carding SR. 2018. Fantastic voyage: the journey of intestinal microbiota-derived microvesicles through the body. Biochem Soc Trans 46:1021–1027. doi:10.1042/BST20180114 30154095 PMC6195637

[154] Brown EM, Temple ER, Jeanfavre S, Avila-Pacheco J, Taylor N, Liu K, Nguyen PNU, Mohamed AMT, Ung P, Walker RA, Graham DB, Clish CB, Xavier RJ. 2025. Bacteroides sphingolipids promote anti-inflammatory responses through the mevalonate pathway. Cell Host & Microbe 33:901–914. doi:10.1016/j.chom.2025.05.007 40449488 PMC12169427

[155] Chatzidaki-Livanis M, Coyne MJ, Comstock LE. 2014. An antimicrobial protein of the gut symbiont Bacteroides fragilis with a MACPF domain of host immune proteins. Mol Microbiol 94:1361–1374. doi:10.1111/mmi.12839 25339613 PMC4262677

[156] Xerri NL, Payne SM. 2022. Bacteroides thetaiotaomicron outer membrane vesicles modulate virulence of shigella flexneri. mBio 13:e0236022. doi:10.1128/mbio.02360-22 36102517 PMC9600379

[157] Rakoff-Nahoum S, Coyne MJ, Comstock LE. 2014. An ecological network of polysaccharide utilization among human intestinal symbionts. Curr Biol 24:40–49. doi:10.1016/j.cub.2013.10.077 24332541 PMC3924574

[158] Stentz R, Horn N, Cross K, Salt L, Brearley C, Livermore DM, Carding SR. 2015. Cephalosporinases associated with outer membrane vesicles released by Bacteroides spp. protect gut pathogens and commensals against β-lactam antibiotics. J Antimicrob Chemother 70:701–709. doi:10.1093/jac/dku466 25433011 PMC4319488

[159] Sartorio MG, Pardue EJ, Scott NE, Feldman MF. 2023. Human gut bacteria tailor extracellular vesicle cargo for the breakdown of diet- and host-derived glycans. Proc Natl Acad Sci USA 120:e2306314120. doi:10.1073/pnas.2306314120 37364113 PMC10319031

[160] Valguarnera E, Scott NE, Azimzadeh P, Feldman MF. 2018. Surface exposure and packing of lipoproteins into outer membrane vesicles are coupled processes in Bacteroides mSphere 3:e00559-18. doi:10.1128/mSphere.00559-18 30404931 PMC6222051

[161] van Doorn J, Oudega B, MacLaren DM. 1992. Characterization and detection of the 40 kDa fimbrial subunit of Bacteroides fragilis BE1. Microb Pathog 13:75–79. doi:10.1016/0882-4010(92)90033-k 1359379

[162] Lee JY, Sojar HT, Bedi GS, Genco RJ. 1991. Porphyromonas (Bacteroides) gingivalis fimbrillin: size, amino-terminal sequence, and antigenic heterogeneity. Infect Immun 59:383–389. doi:10.1128/iai.59.1.383-389.1991 1987052 PMC257752

[163] Dickinson DP, Kubiniec MA, Yoshimura F, Genco RJ. 1988. Molecular cloning and sequencing of the gene encoding the fimbrial subunit protein of Bacteroides gingivalis. J Bacteriol 170:1658–1665. doi:10.1128/jb.170.4.1658-1665.1988 2895100 PMC211014

[164] Yoshimura F, Takahashi Y, Hibi E, Takasawa T, Kato H, Dickinson DP. 1993. Proteins with molecular masses of 50 and 80 kilodaltons encoded by genes downstream from the fimbrilin gene (fimA) are components associated with fimbriae in the oral anaerobe Porphyromonas gingivalis. Infect Immun 61:5181–5189. doi:10.1128/iai.61.12.5181-5189.1993 7901164 PMC281299

[165] Macadangdang BR, Wang Y, Woodward C, Revilla JI, Shaw BM, Sasaninia K, Makanani SK, Berruto C, Ahuja U, Miller JF. 2024 Targeted protein evolution in the gut microbiome by diversity-generating retroelements. bioRxiv. doi:10.1101/2024.11.15.621889 PMC12787319 41066555

[166] Bayley DP, Rocha ER, Smith CJ. 2000. Analysis of cepA and other Bacteroides fragilis genes reveals a unique promoter structure. FEMS Microbiol Lett 193:149–154. doi:10.1016/S0378-1097(00)00472-9 11094294

[167] Chen S, Bagdasarian M, Kaufman MG, Bates AK, Walker ED. 2007. Mutational analysis of the ompA promoter from Flavobacterium johnsoniae. J Bacteriol 189:5108–5118. doi:10.1128/JB.00401-07 17483221 PMC1951883

[168] Hunnicutt DW, McBride MJ. 2001. Cloning and characterization of the Flavobacterium johnsoniae gliding motility genes gldD and gldE. J Bacteriol 183:4167–4175. doi:10.1128/JB.183.14.4167-4175.2001 11418556 PMC95305

[169] Vingadassalom D, Kolb A, Mayer C, Collatz E, Podglajen I. 2007. Probing the importance of selected phylum-specific amino acids in sigma A of Bacteroides fragilis, a primary sigma factor naturally devoid of an N-terminal acidic region 1.1. J Biol Chem 282:3442–3449. doi:10.1074/jbc.M608855200 17150963

[170] Vingadassalom D, Kolb A, Mayer C, Rybkine T, Collatz E, Podglajen I. 2005. An unusual primary sigma factor in the Bacteroidetes phylum. Mol Microbiol 56:888–902. doi:10.1111/j.1365-2958.2005.04590.x 15853878

[171] Rocha E R, Selby T, Coleman JP, Smith CJ. 1996. Oxidative stress response in an anaerobe, Bacteroides fragilis: a role for catalase in protection against hydrogen peroxide. J Bacteriol 178:6895–6903. doi:10.1128/jb.178.23.6895-6903.1996 8955312 PMC178591

[172] Herren CD, Rocha ER, Smith CJ. 2003. Genetic analysis of an important oxidative stress locus in the anaerobe Bacteroides fragilis. Gene 316:167–175. doi:10.1016/s0378-1119(03)00759-5 14563563

[173] Rocha ER, Owens G, Smith CJ. 2000. The redox-sensitive transcriptional activator OxyR regulates the peroxide response regulon in the obligate anaerobe Bacteroides fragilis. J Bacteriol 182:5059–5069. doi:10.1128/JB.182.18.5059-5069.2000 10960088 PMC94652

[174] Sund CJ, Rocha ER, Tzianabos AO, Wells WG, Gee JM, Reott MA, O’Rourke DP, Smith CJ. 2008. The Bacteroides fragilis transcriptome response to oxygen and H2O2: the role of OxyR and its effect on survival and virulence. Mol Microbiol 67:129–142. doi:10.1111/j.1365-2958.2007.06031.x 18047569

[175] Betteken MI, Rocha ER, Smith CJ. 2015. Dps and DpsL mediate survival in vitro and in vivo during the prolonged oxidative stress response in Bacteroides fragilis. J Bacteriol 197:3329–3338. doi:10.1128/JB.00342-15 26260459 PMC4573723

[176] Teixeira FL, Pauer H, Costa SB, Smith CJ, Domingues RMCP, Rocha ER, Lobo LA. 2018. Deletion of BmoR affects the expression of genes related to thiol/disulfide balance in Bacteroides fragilis. Sci Rep 8:14405. doi:10.1038/s41598-018-32880-7 30258073 PMC6158253

[177] Baughn AD, Malamy MH. 2004. The strict anaerobe Bacteroides fragilis grows in and benefits from nanomolar concentrations of oxygen. Nature 427:441–444. doi:10.1038/nature02285 14749831

[178] García-Bayona L, Coyne MJ, Hantman N, Montero-Llopis P, Von SS, Ito T, Malamy MH, Basler M, Barquera B, Comstock LE. 2020. Nanaerobic growth enables direct visualization of dynamic cellular processes in human gut symbionts. Proc Natl Acad Sci USA 117:24484–24493. doi:10.1073/pnas.2009556117 32938803 PMC7533675

[179] Ito T, Gallegos R, Matano LM, Butler NL, Hantman N, Kaili M, Coyne MJ, Comstock LE, Malamy MH, Barquera B. 2020. Genetic and biochemical analysis of anaerobic respiration in Bacteroides fragilis and its importance in vivo mBio 11:e03238-19. doi:10.1128/mBio.03238-19 32019804 PMC7002350

[180] Butler NL, Ito T, Foreman S, Morgan JE, Zagorevsky D, Malamy MH, Comstock LE, Barquera B. 2023. Bacteroides fragilis maintains concurrent capability for anaerobic and nanaerobic respiration. J Bacteriol 205:e0038922. doi:10.1128/jb.00389-22 36475831 PMC9879120

[181] Albenberg L, Esipova TV, Judge CP, Bittinger K, Chen J, Laughlin A, Grunberg S, Baldassano RN, Lewis JD, Li H, Thom SR, Bushman FD, Vinogradov SA, Wu GD. 2014. Correlation between intraluminal oxygen gradient and radial partitioning of intestinal microbiota. Gastroenterology 147:1055–63. doi:10.1053/j.gastro.2014.07.020 25046162 PMC4252572

[182] Li J, Gálvez EJC, Amend L, Almási É, Iljazovic A, Lesker TR, Bielecka AA, Schorr E-M, Strowig T. 2021. A versatile genetic toolbox for Prevotella copri enables studying polysaccharide utilization systems. EMBO J 40:e108287. doi:10.15252/embj.2021108287 34676563 PMC8634118

[183] Shoemaker NB, Getty C, Gardner JF, Salyers AA. 1986. Tn4351 transposes in Bacteroides spp. and mediates the integration of plasmid R751 into the Bacteroides chromosome. J Bacteriol 165:929–936. doi:10.1128/jb.165.3.929-936.1986 3005243 PMC214518

[184] Smith CJ. 1985. Development and use of cloning systems for Bacteroides fragilis: cloning of a plasmid-encoded clindamycin resistance determinant. J Bacteriol 164:294–301. doi:10.1128/jb.164.1.294-301.1985 2995313 PMC214243

[185] Guthrie EP, Salyers AA. 1986. Use of targeted insertional mutagenesis to determine whether chondroitin lyase II is essential for chondroitin sulfate utilization by Bacteroides thetaiotaomicron. J Bacteriol 166:966–971. doi:10.1128/jb.166.3.966-971.1986 3011755 PMC215219

[186] Salyers AA, Guthrie EP. 1988. A deletion in the chromosome of Bacteroides thetaiotaomicron that abolishes production of chondroitinase II does not affect survival of the organism in gastrointestinal tracts of exgermfree mice. Appl Environ Microbiol 54:1964–1969. doi:10.1128/aem.54.8.1964-1969.1988 3140726 PMC202787

[187] Tang YP, Malamy MH. 2000. Isolation of Bacteroides fragilis mutants with in vivo growth defects by using Tn4400’, a modified Tn4400 transposition system, and a new screening method. Infect Immun 68:415–419. doi:10.1128/IAI.68.1.415-419.2000 10603420 PMC97153

[188] Wu M, McNulty NP, Rodionov DA, Khoroshkin MS, Griffin NW, Cheng J, Latreille P, Kerstetter RA, Terrapon N, Henrissat B, Osterman AL, Gordon JI. 2015. Genetic determinants of in vivo fitness and diet responsiveness in multiple human gut Bacteroides. Science 350:aac5992. doi:10.1126/science.aac5992 26430127 PMC4608238

[189] Liu H, Shiver AL, Price MN, Carlson HK, Trotter VV, Chen Y, Escalante V, Ray J, Hern KE, Petzold CJ, Turnbaugh PJ, Huang KC, Arkin AP, Deutschbauer AM. 2021. Functional genetics of human gut commensal Bacteroides thetaiotaomicron reveals metabolic requirements for growth across environments. Cell Rep 34:108789. doi:10.1016/j.celrep.2021.108789 33657378 PMC8121099

[190] Mimee M, Tucker AC, Voigt CA, Lu TK. 2015. Programming a human commensal bacterium, Bacteroides thetaiotaomicron, to sense and respond to stimuli in the murine gut microbiota. Cell Syst 1:62–71. doi:10.1016/j.cels.2015.06.001 26918244 PMC4762051

[191] Lim B, Zimmermann M, Barry NA, Goodman AL. 2017. Engineered regulatory systems modulate gene expression of human commensals in the gut. Cell 169:547–558. doi:10.1016/j.cell.2017.03.045 28431252 PMC5532740

[192] García-Bayona L, Comstock LE. 2019. Streamlined genetic manipulation of diverse Bacteroides and Parabacteroides isolates from the human gut microbiota. MBio 10:e01762-19. doi:10.1128/mBio.01762-19 31409684 PMC6692515

[193] Bencivenga-Barry NA, et al.. 2020. Genetic manipulation of wild human gut bacteroides. J Bacteriol 202. doi:10.1128/JB.00544-19 PMC6964735 31712278

[194] Zheng L, Tan Y, Hu Y, Shen J, Qu Z, Chen X, Ho CL, Leung EL-H, Zhao W, Dai L. 2022. CRISPR/Cas-based genome editing for human gut commensal Bacteroides Species. ACS Synth Biol 11:464–472. doi:10.1021/acssynbio.1c00543 34990118

[195] Feng J, Qian Y, Zhou Z, Ertmer S, Vivas EI, Lan F, Hamilton JJ, Rey FE, Anantharaman K, Venturelli OS. 2022. Polysaccharide utilization loci in Bacteroides determine population fitness and community-level interactions. Cell Host Microbe 30:200–215. doi:10.1016/j.chom.2021.12.006 34995484 PMC9060796

[196] Liang J, Tan Y. 2023. Highly efficient CRISPR-mediated base editing for the gut Bacteroides spp. with pnCasBS-CBE. Biotechnol J 18:e2200504. doi:10.1002/biot.202200504 37010073

[197] Beller ZW, Wesener DA, Seebeck TR, Guruge JL, Byrne AE, Henrissat S, Terrapon N, Henrissat B, Rodionov DA, Osterman AL, Suarez C, Bacalzo NP Jr, Chen Y, Couture G, Lebrilla CB, Zhang Z, Eastlund ER, McCann CH, Davis GD, Gordon JI. 2023. Inducible CRISPR-targeted “knockdown” of human gut Bacteroides in gnotobiotic mice discloses glycan utilization strategies. Proc Natl Acad Sci USA 120:e2311422120. doi:10.1073/pnas.2311422120 37733741 PMC10523453

[198] Prezza G, Westermann AJ. 2024. CRISPR interference-based functional small RNA genomics. Methods Mol Biol 2741:101–116. doi:10.1007/978-1-0716-3565-0_6 38217650

[199] Hayashi N, Lai Y, Fuerte-Stone J, Mimee M, Lu TK. 2024. Cas9-assisted biological containment of a genetically engineered human commensal bacterium and genetic elements. Nat Commun 15:2096. doi:10.1038/s41467-024-45893-w 38453913 PMC10920895

[200] Kang M, Kim K, Cho BK. 2024. CRISPRi-driven genetic screening for designing novel microbial phenotypes. Methods Mol Biol 2760:117–132. doi:10.1007/978-1-0716-3658-9_7 38468085

[201] Schnizlein MK, Dubey AA, Fiebig A, Crosson S. 2025. Genetic- and culture-based tools for studying Bacteroides fragilis Microbiol Resour Announc 14:e0000625. doi:10.1128/mra.00006-25 40130927 PMC12060699

